# Simultaneous and independent capture of multiple Rayleigh dielectric nanospheres with sine-modulated Gaussian beams

**DOI:** 10.1038/s41598-020-80470-3

**Published:** 2021-01-08

**Authors:** Jingjing Su, Nan Li, Xianfan Wang, Xingfan Chen, Huizhu Hu

**Affiliations:** 1 State Key Laboratory of Modern Optical Instrumentation, College of Optical Science and Engineering, Zhejiang University, Hangzhou, 310027 China; 2 Quantum Sensing Center, Zhejiang Lab, Hangzhou, 310000 China

**Keywords:** Physics, Optical physics

## Abstract

This study investigates the propagation properties and radiation forces on Rayleigh dielectric particles produced by novel sine-modulated Gaussian beams (SMGBs) because of the unique focusing properties of four independent light intensity distribution centers and possessing many deep potential wells in the output plane of the target laser. The described beams can concurrently capture and manipulate multiple Rayleigh dielectric spheres with high refractive indices without disturbing each other at the focus plane. Spheres with a low refractive index can be guided or confined in the focus but cannot be stably trapped in this single beam trap. Simulation results demonstrate that the focused SMGBs can be used to trap particle in different planes by increasing the sine-modulate coefficient *g*. The conditions for effective and stable capture of high-index particles and the threshold of detectable radius are determined at the end of this study.

## Introduction

The optical trapping technique or optical tweezer has been intensively applied in atomic optics, optical communication and manipulation, and biotechnology^1–5^. Since Ashkin et al. first demonstrated that a focused Gaussian beam could trap particles^6–8^, providing a useful method for trapping micron particles, optical tweezers have made great progress in many fields. Through the unremitting efforts of researchers, various beams were reported and used for trapping and manipulating tiny objects, including Bessel–Gaussian beams^9–11^, hollow Gaussian beams^12–14^, partially coherent beams^15,16^, and airy beams^17–19^. The Bessel beam is well-known for its non-diffractive and self-reconstruction characteristics, and it can axially transport particles and simultaneously capture multilayer particles^9^. Kuga et al. constructed a novel hollow laser beam trap to capture neutral atoms^20^. Because the energy at the focal point of the dark hollow beam is low, the Brownian motion of the particles at the focal point can be effectively reduced, improving trapping efficiency. Partially coherent beams, such as modified Bessel–Gaussian and elegant Laguerre–Gaussian beams, can simultaneously capture particles with high and low refractive indices with a suitable mode order, conducted both in theory and experiment^16,21^. Circular airy beams show that the focus position of the blocked beams remains the same, enhancing its abrupt autofocusing property^18^. Applying these beams has improved the capture range and vertical depth as opposed to the Gaussian light field. However, single optical tweezers that can simultaneously and independently operate multiple particles remains to be urgently resolved.

Holographic optical tweezers can modify the wave front of the input beams and transform them into entire classes of optical traps, which has been widely studied^22,23^. For example, Laguerre–Gaussian beams can rotate microspheres after focusing^24^, and airy beams with a light clearing function can travel along a parabola trajectory^25,26^. Holographic optical tweezers can also control multiple particles at multiple positions and angles in real-time^27^. In 1998, Dufresne et al. demonstrated the feasibility of creating multiple optical tweezers from a single laser beam using diffractive optical elements^28^. Curtis et al. used the spatial light modulator to generate 20 × 20 arrays of traps^29^. In 1997, Casperson demonstrated that sinusoidal-Gaussian beams are another solution to propagate electromagnetic waves in free space. Placing a grating with a periodic transverse amplitude transmission profile can yield a transformation profile that could convert the input beam into a sinusoidal-Gaussian beam^30^. To the best of our knowledge, the radiation force of sine-modulated Gaussian beams (SMGBs) on the Rayleigh particle has not been studied before.

This study proposes an SMGB that can generate four light intensity distribution centers to independently trap the focal plane up to four high refractive index Rayleigh dielectric nanospheres. Using the well-known Collins integration and ABCD transformation matrix, the electrical field profile, gradient force, and scattering force of the focused SMGBs are theoretically calculated. Several numerical simulations are performed to investigate the relationship between the longitudinal gradient force and the sine-modulation coefficient *g*. The equilibrium points increase by changing the parameter *g*. Furthermore, the stability criterion and the Boltzmann factor of SMGBs are obtained. The comparison of the radiation and Brownian forces shows that SMGBs can trap Rayleigh dielectric particles with a radius close to 4.431 nm. This novel class of waves can exhibit unique light force characteristics and provide advantages in research areas such as biology, physics, and chemistry^31,32^.

## Propagation of SMGBs through an ABCD system

In the rectangular coordinate system, the electric field of SMGBs at the origin plane (${z}_{1}=0$) takes the form

1 $$ E_{1} (x_{1} ,y_{1} ,0) = E_{0} \exp \left( {\frac{{ - ik(x_{1}^{2} + y_{1}^{2} )}}{{2q_{1} }}} \right)\sin \left( {\frac{{gx_{1} }}{{w_{0} }}} \right)\sin \left( {\frac{{gy_{1} }}{{w_{0} }}} \right), $$

where ${E}_{0}$ denotes a constant related to the laser beams power *P*. *q*_1_ is a complex parameter of the incident Gaussian beam, *g* is the modulation coefficient related to the sine and can take on any value other than zero, and ${w}_{0}$ represents the waist size of the corresponding normal Gaussian beam.

Using the extended Huygens–Fresnel diffraction integral in the paraxial approximation, we can determine the electric field of SMGBs using an ABCD optical system

2 $$ \begin{aligned} E_{2} (x,y,{\text{z}}) & = \frac{i}{\lambda B}\exp ( - ikz)\iint {dx_{1} dy_{1} E_{1} (x_{1} ,y_{1} ,0)} \\ & \quad \exp \left\{ { - \frac{ik}{{2B}}\left[ {A(x_{1}^{2} + y_{1}^{2} ) - 2(xx_{1} + yy_{1} ) + D(x^{2} + y^{2} )} \right]} \right\}. \\ \end{aligned} $$

**Figure 1:**
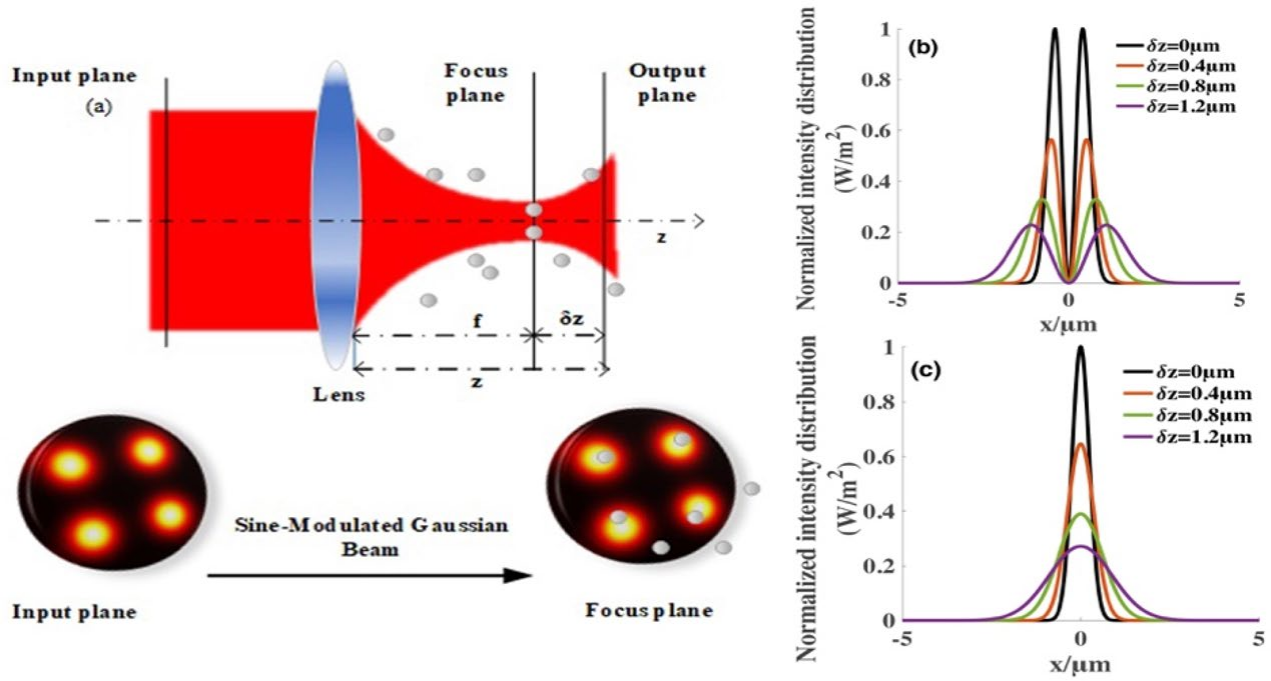
An illustration of the focusing optical system. (**a**) shows the schematic of the SMGBs, where $z$ is the longitudinal coordinate at the beginning of the focusing lens, $z=f +\delta z.$ $\delta z$ is the distance from the focal point on the axis, and $f$ is the focal length of the thin lens. (**b,c**) demonstrate the normalized intensity distribution of SMGBs and Gaussian beams at different distance values of $\delta z$, respectively. Other parameters are $\lambda =1.064 \,\upmu{\mathrm{m}}$, ${w}_{0}= 5\,{\mathrm{mm}}$*,*$f=5\,{\mathrm{mm}}$, $P=4\,{\mathrm{w}}$ and the sine-modulation coefficient $g=1$.

We probe the focusing properties of SMGBs by considering the beam propagation through a lens system (Fig. 1). The transfer matrix for this system is given by the reference^33^, where $z$ is the longitudinal coordinate at the beginning of the focusing lens, $z=f +\delta z$*.* $\delta z$ is the distance from the focal point on the axis, and $f$ is the focal length of the thin lens. We assume that $P$ is the incident light power. Therefore, we can also obtain the initial value of the electric field.

3 $$ \left( {\begin{array}{*{20}c} A & B \\ C & D \\ \end{array} } \right) = \left( {\begin{array}{*{20}c} 1 & z \\ 0 & 1 \\ \end{array} } \right)\left( {\begin{array}{*{20}c} 1 & 0 \\ { - {\raise0.7ex\hbox{$1$} \!\mathord{\left/ {\vphantom {1 f}}\right.\kern-\nulldelimiterspace} \!\lower0.7ex\hbox{$f$}}} & 1 \\ \end{array} } \right) = \left( {\begin{array}{*{20}c} {1 - {\raise0.7ex\hbox{$z$} \!\mathord{\left/ {\vphantom {z f}}\right.\kern-\nulldelimiterspace} \!\lower0.7ex\hbox{$f$}}} & z \\ { - {\raise0.7ex\hbox{$1$} \!\mathord{\left/ {\vphantom {1 f}}\right.\kern-\nulldelimiterspace} \!\lower0.7ex\hbox{$f$}}} & 1 \\ \end{array} } \right), $$

4 $$ E_{0}^{2} = \frac{4P}{{\pi w_{0}^{2} n_{m} \varepsilon_{0} c}}\left( {\frac{1}{{1 - \exp ( - g^{2} /2)}}} \right)^{2} . $$

By substituting Eqs. (1), (3), and (4) into Eq. (2) and after straightforward integration, we can obtain

5 $$ \begin{aligned} E_{2} (x,y,{\text{z}}) & = E_{0} \exp ( - ikz)\frac{{q_{1} }}{{Aq_{1} + B}}\exp \left( {\frac{{ - ik(x^{2} + y^{2} )}}{2}\left( {\frac{{C + D/q_{1} }}{{A + B/q_{1} }}} \right)} \right) \\ & \quad \exp \left( {\frac{{i\alpha^{2} B}}{k}\frac{{q_{1} }}{{Aq_{1} + B}}} \right)\sin \left( {\frac{gx}{{w_{0} }}\frac{{q_{1} }}{{Aq_{1} + B}}} \right)\sin \left( {\frac{gy}{{w_{0} }}\frac{{q_{1} }}{{Aq_{1} + B}}} \right). \\ \end{aligned} $$

where $$q_{1} = \frac{{i\pi w_{0}^{2} }}{\lambda }$$.

Figure 1 shows the intensity distribution of SMGBs through a lens system to visualize the profiles of SMGBs. Figure 1a illustrates the focusing optical system of SMGBs with four independent focus points at both the initial and focal planes. Figure 1b,c describe the dependence of the intensity distribution of the SMGBs and Gaussian beams on the beam index $\delta z$, respectively, further examining the SMGBs’ focusing properties in detail. Both Gaussian beams and SMGBs’ intensity amplitudes decrease with the increase in the distance from the focal point $\delta z$. Gaussian beams have only one light intensity center in one direction, whereas SMGBs has two. Because of the symmetrical distribution of SMGBs in the focal plane, we could have four independent potential wells of the target laser. As the distance $\delta z$ of SMGBs decreases, the dark area in the middle increases and the four light centers move further from the focus point. Because of this special focusing characteristic, we assume that SMGBs can trap multiple particles with different refractive indices at the focal plane.

## Optical forces on a Rayleigh dielectric sphere produced by SMGBs

This section demonstrates the radiation forces exerted on a nanosphere produced by SMGBs. Since the radius of Rayleigh’s sphere is much smaller than the light wavelength $(a \le \lambda /20)$, it is the electric dipole of the light field. Here, according to Harada and Asakura, two types of optical forces act on the sphere: the scattering force ${F}_{Scat}$ and the gradient force ${F}_{Grad}$. The scattering force arises from the light scattering by the dipole and travels along the beam propagation direction, which is proportional to the intensity of the beam. And the nonuniform electromagnetic field on the dipole that acts as the restoring forces responsible for drawing the particles back to the beam center produces the gradient. The scattering and the gradient force are defined by^34,35^

6 $$ \mathop F\limits^{ \to }_{Scat} (x,y,z) = \mathop {e_{z} }\limits^{ \to } \frac{{n_{m}^{2} \varepsilon_{0} }}{2}C_{pr} \left| {E(x,y,z)} \right|^{2} , $$

7 $$ \mathop F\limits^{ \to }_{Grad} (x,y,z) = \pi n_{m}^{2} \varepsilon_{0} a^{3} \left( {\frac{{n_{r}^{2} - 1}}{{n_{r}^{2} + 2}}} \right)\nabla \left| {\mathop E\limits^{ \to } (x,y,z)} \right|^{2} , $$

with

8 $$ C_{pr} = C_{scat} = \frac{8}{3}\pi (ka)^{4} a^{2} \left( {\frac{{n_{r}^{2} - 1}}{{n_{r}^{2} + 2}}} \right)^{2} . $$

where $\overrightarrow{{e}_{z}}$ is a unit vector in the beam propagation direction, $c$ is the speed of the light field in a vacuum, and ${C}_{pr}$ equals the scattering cross section ${C}_{Scat}$ for a dielectric sphere in the Rayleigh regime. In the following simulations, we select the radius of nanosphere as $a=20\,{\mathrm{nm}}$ which is within the Rayleigh range for the wavelength we consider. ${n}_{r}={n}_{p}/{n}_{m}$ is a relative index with ${n}_{p}$ and ${n}_{m}$, respectively, representing the refractive index of the sphere as ${n}_{p}=1$ (air bubble), ${n}_{p}=1.59$ (glass), and ambient as ${n}_{m}=1.33$ (water)^36^.

**Figure 2:**
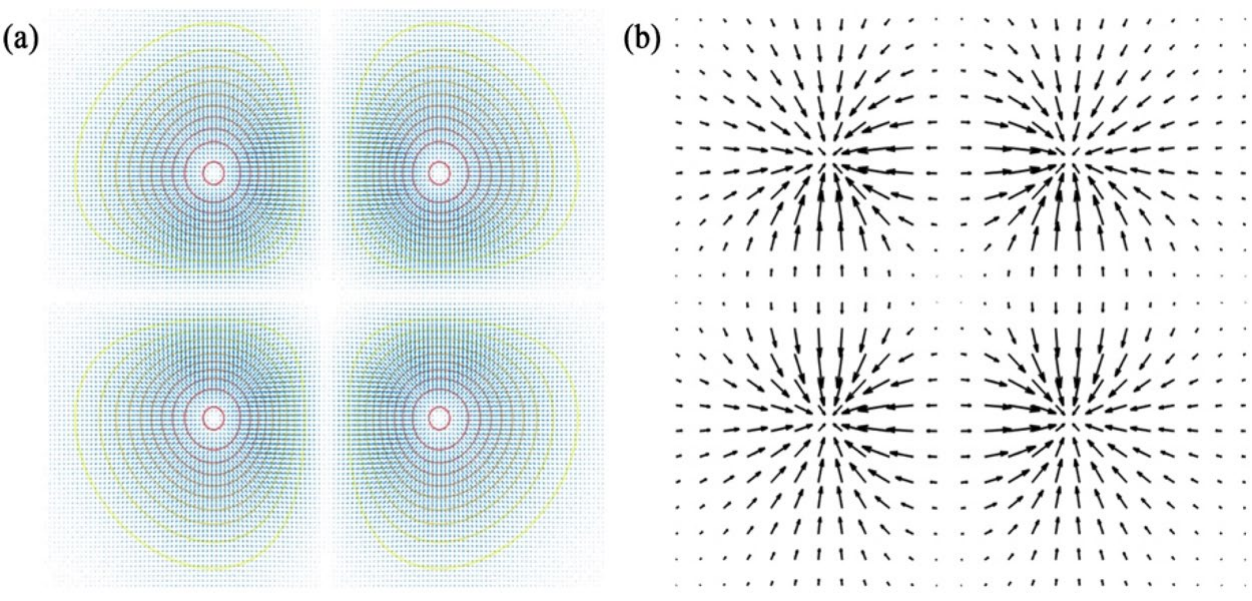
(**a**) Contour plot of the intensity and (**b**) gradient field of SMGBs in the plane perpendicular to the axis of propagation. The colors represent the magnitudes of radiation forces in (**a**). The directions and lengths of the black arrows in (**b**) represent the directions and magnitudes of the resultant forces.

Figure 2 shows the simulated summation of the radiation force field exerted on the nanospheres at the focal plane. The gradient arrows of the gradient field graph are toward the centers, whereas the directions and lengths of the arrows represent the directions and magnitudes of the resultant forces (Fig. 2b). The position of trapped particles slightly deviates from the equilibrium point. The gradient force is proportional to the intensity gradient and points to the focus, which is the direction of the intensity gradient maximum. However, the gradient force is nearly zero at the focus point (Fig. 2). The scattering force is proportional to the optical intensity and points toward beam propagation. In the focus point, the gradient force is small; therefore, the optimum position for the trapped particles is slightly shifted from the focus. Therefore, in the following article, we also consider the influence of scattering at different planes. Because of the four focal points in the intensity distribution from the contour plot in Fig. 2a, SMGBs can independently and simultaneously trap and manipulate multiple particles over other types of beams.

**Figure 3:**
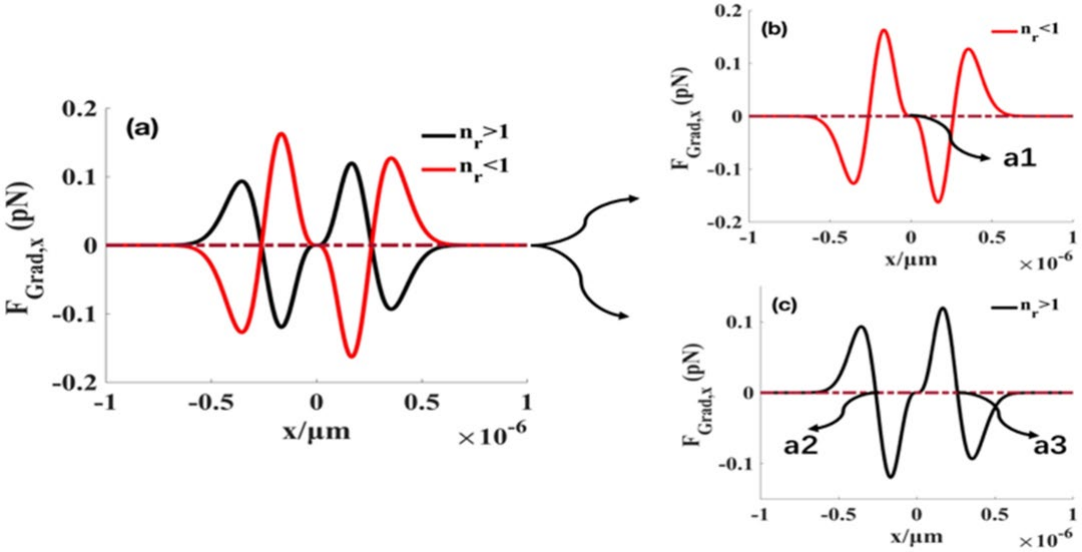
Transverse gradient forces produced by focused SMGBs on high-index $({n}_{p}=1.592)$ and low-index $({n}_{p}=1)$ particles along $x$ direction. The black solid curve shows particles with ${n}_{p}=1.592$, and the red solid curve shows particles with ${n}_{p}=1$. The reddish-brown dotted line is the coordinate axes. (**a**) Transverse gradient force at the focus plane. The exploded diagram of (**a**) is (**b**) and (**c**). We select a sphere with a radius $a=20 \,\mathrm{nm},$ and ${n}_{r}={n}_{p}/{n}_{m}$ represents the relative refractive index. ${n}_{m}=1.332$ is the refractive index of the surrounding field, and the high and low refractive indices are the homogeneous Rayleigh nanospheres. Other parameters are $\lambda =1.064\,\upmu{\mathrm{m}}$, ${w}_{0}= 5\,{\mathrm{mm}}$*,*$f=5 \,{\mathrm{mm}}$, $P=4\,{\mathrm{w}}$, and the sine-modulation coefficient $g=1$.

**Figure 4:**
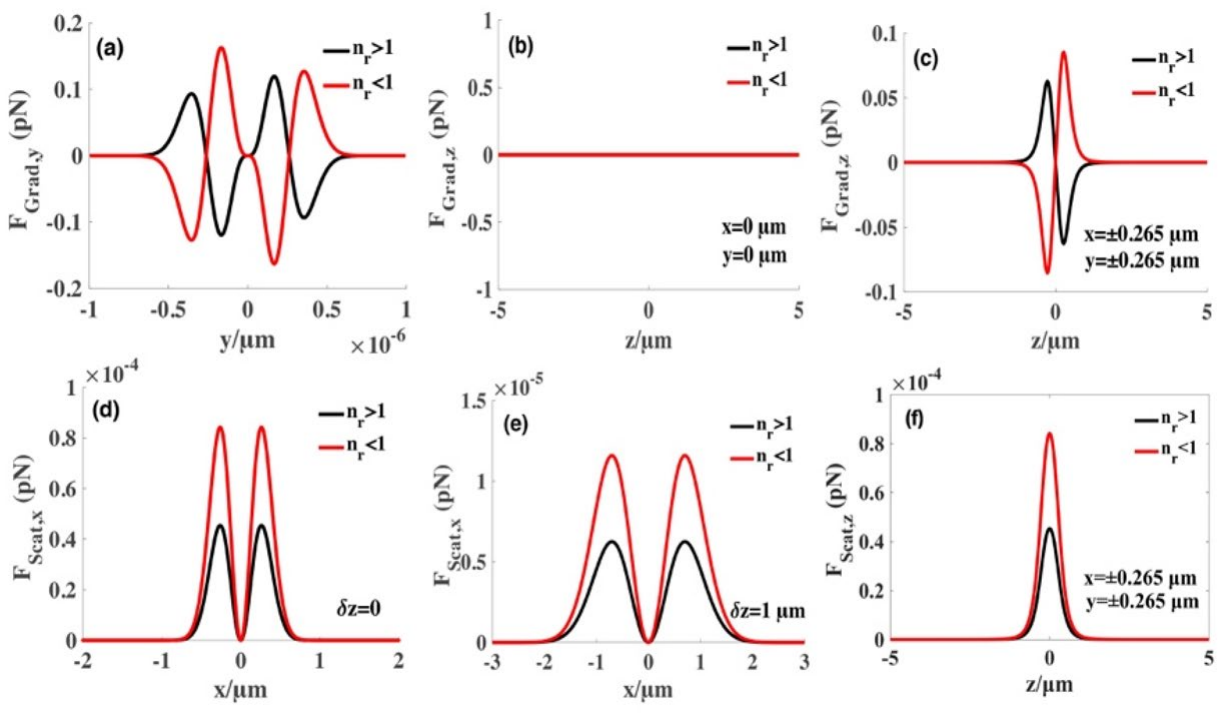
Radiation forces produced by focused SMGBs on high-index (*n*_*p*_ = 1.592*)* and low-index (*n*_*p*_ = 1) particles. (**a**) Transverse gradient force at the focus plane along the $y$ direction. (**b**) Longitudinal gradient force exerted on Rayleigh dielectric spheres at *x *= 0 µm, $y\hspace{0.17em}=\hspace{0.17em}0\,\upmu{\mathrm{m}}$. (**c**) Longitudinal gradient force exerted on Rayleigh dielectric spheres at $x=\pm 0.265 \,\upmu{\mathrm{m}}, y=\pm 0.265\,\upmu{\mathrm{m}}$. (**d**,**e**) The transverse scattering force produced by SMGBs at different planes. (**f**) Longitudinal gradient force exerted on Rayleigh dielectric spheres at $x=\pm 0.265\,\upmu{\mathrm{m}}, y=\pm 0.265 \,\upmu{\mathrm{m}}$. We select a sphere with a radius $a=20 \,\mathrm{nm}$ and ${n}_{r}={n}_{p}/{n}_{m}$ to represent the relative refractive index. ${n}_{m}=1.332$ is the refractive index of the surrounding field, and the high and low refractive indices are the homogeneous Rayleigh nanospheres. Other parameters are $\lambda =1.064 \,\upmu{\mathrm{m}}$, ${w}_{0}= 5 \,{\mathrm{mm}}$*,*$f=5\, \mathrm{mm}$, $P=4 \,\mathrm{w}$, and the sine-modulation coefficient $g=1$.

Based on the theoretical analysis above, the gradient and scattering forces are calculated (Figs. 3, 4). The sign of the gradient force indicates the direction of the force. The transverse gradient force for positive ${F}_{Grad,x}$ is along the $+x$ direction and that for negative ${F}_{Grad,x}$ is along the $-x$ direction. Similarly, for positive (negative) ${F}_{Grad,z}$, the longitudinal gradient force is in the $+z (-z)$ direction. The scattering force is always along the $+z$ direction.

In Fig. 3, we depict the transverse gradient forces along the $x$ direction. Figure 3b,c are the exploded views of Fig. 3a. Figure 3b shows that a stable equilibrium point ${a}_{1}$ toward the transverse gradient force exists for particles with a low refractive index. However, high refractive index particles have two stable equilibrium points ${a}_{2}$ and ${a}_{3}$ at $x=\pm 0.265 \,\upmu{\mathrm{m}}$. From Figs. 3a,c, 4c,f, one can find that the high-index particles have four stable equilibrium points $(x=\pm 0.265 \,\upmu{\mathrm{m}},y=\pm 0.265 \,\upmu{\mathrm{m}})$ in the focal plane. Note that multiple stable equilibrium points exist for high-index nanospheres. Therefore, the results show that the focused SMGBs can trap or manipulate multiple particles.

Figures 3a,b and 4a,c show that low-index particles have one stable equilibrium point at the focus in the transverse direction, whereas Fig. 4b shows a red channel along the z-axis in the intensity distribution, indicating that the axial gradient force always equals zero when $x=0,y=0$. Therefore, low-index particles can be confined or guided in the focus (2D trap) but cannot be stably trapped in a single SMGB. We can add two orthogonal laser beams to form a three-dimension trap to stably trap the particles. Figure 4d–f show the scattering force at different propagation distances from the focus point. We find that the scattering force is negligible compared to the transverse and longitudinal gradient forces (Figs. 3a, 4a,c).

**Figure 5:**
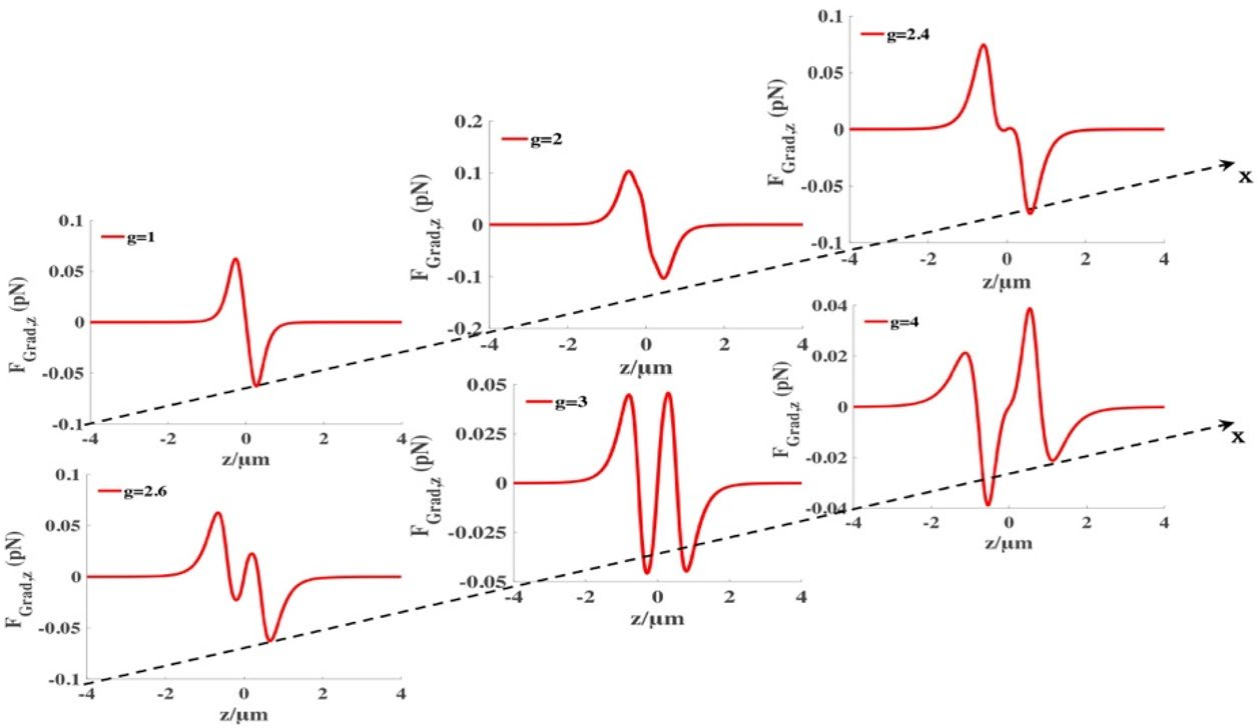
Longitudinal gradient force produced by focused SMGBs on the high-index $\left({n}_{p}=1.592\right)$ Rayleigh dielectric spheres at (*x*, *y*) = (0.265, 0.265) µm with different sine-modulation coefficient $g$. We select a sphere with a radius $a=20\, \mathrm{nm},$ the other parameters are $\lambda =1.064 \,\upmu{\mathrm{m}}$, ${w}_{0}= 5 \,\mathrm{mm}$*,*$f=5 \,\mathrm{mm}$ and $P=4 \,\mathrm{w}$.

In Fig. 5, we plot the changes of the longitudinal gradient forces of the high-index particles at one of the equilibrium points $(x=0.265\,\upmu{\mathrm{m}}, y=0.265 \,\upmu{\mathrm{m}}$) of the focal plane with different sine-modulation coefficient $g$. From the longitudinal gradient forces, one can find that there is one stable equilibrium at $g=1$, as $g$ increases, it is observed that there appear two stable equilibrium points. In this case, we can use the focused SMGBs to trap spheres in different plane.

**Figure 6:**
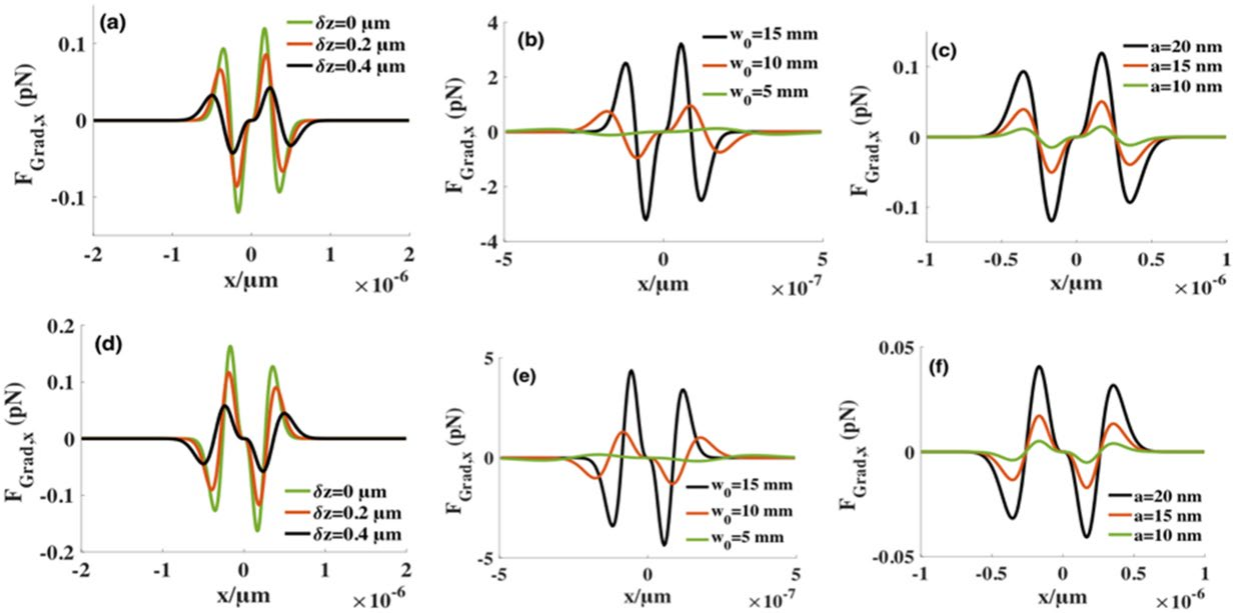
The changes of the transverse gradient forces for several values on high-index and low-index spheres, respectively. For (**a**) and (**d**), the beam waist ${w}_{0}= 5 \,\mathrm{mm}$ and particle radius $a=20 nm$. For (**b**) and (**e**), particle radius *a* = 20 nm and the distance $\delta z=0$ from the focus point*.* For (**c**) and (**f**), the distance $\delta z=0$ from the focus point and the beam waist ${w}_{0}= 5 \,\mathrm{mm}$. Other parameters are $\lambda =1.064 \,\upmu{\mathrm{m}}$, $g=1$*,*$f=5 \,\mathrm{mm}$, and $P=4 \,\mathrm{w}$*.*

To further study the influences of beams’ optical parameters generated by SMGBs on the transverse gradient force, Fig. 6a–c illustrate the radiation forces exerted on the high-index particles for different values of radius $a$, the beam waist ${w}_{0}$, and the distance $\delta z$ from the focus point*.* Figure 6d–f demonstrate the radiation forces exerted on the low-index microspheres. The numerical results show that the gradient force ${F}_{Grad}$ is related to the beam waist ${w}_{0}$, and ${F}_{Grad}$ increases by orders of magnitude as the beam waist ${w}_{0}$ increases from $5 \,\mathrm{mm}$ to 1 $5 \,\mathrm{mm}$. However, ${F}_{Grad}$ is inversely proportional to the distance $\delta z$ between the particle and focal plane. And the gradient potential well is wider with an increase in distance $\delta z.$ The gradient force increases as the particle radius increases. Figure 6a–c show that there are two stable equilibrium points in the focus plane for the high-index particles. In Fig. 6d–f, the trend of the gradient force of changes of the low-index particle is the same as the high-index particles.

## Trapping stability analysis

The above discussion confirms that the radiation forces produced by SMGBs can trap and manipulate Rayleigh dielectric particles of ${n}_{r}>1$. To stably trap spheres under the Rayleigh scattering regime, two conditions must be considered. The potential well of the gradient force should be deep enough to overcome the kinetic energy contributed by Brownian motion because of the thermal fluctuation in the surrounding environment. This stability condition is represented by the Boltzmann factor^6,34^

9 $$ R_{thermal} = \exp ( - U_{\max } /k_{B} T) < < 1, $$

where ${k}_{B}$ is the Boltzmann constant and $T=300 K$ is adopted as the absolute temperature of the particle environment. ${U}_{max}$ is the maximum depth of the potential well, which is written as^5^

10 $$ U_{\max } = \pi \varepsilon_{0} n_{m}^{2} a^{3} \left| {\frac{{n_{r}^{2} - 1}}{{n_{r}^{2} + 2}}} \right|\left| {\vec{E}} \right|_{\max }^{2} . $$

For the high refractive index particles ${(n}_{p}=1.59)$, where the sine-modulation coefficient $g=1$ at one of the maximum intensity position $\left(x=0.265\,\upmu{\mathrm{m}},y=0.265\,\upmu{\mathrm{m}},\delta z=0\right)$ and ${R}_{thermal}=0.0012$, the value of ${R}_{thermal}$ at $g=4$ is about ${R}_{thermal}=0.0197$. The Boltzmann factor values around the focal plane are sufficiently small. Therefore, the particles can overcome Brownian motion and stably captured by SMGBs.

**Figure 7:**
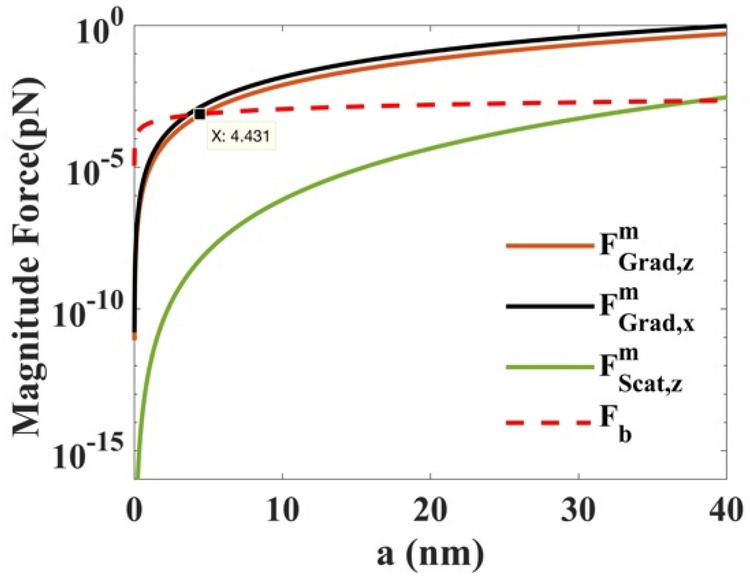
Comparison of ${F}_{Grad,z}^{m}$,${F}_{Scat,x}^{m}$,${F}_{Grad,x}^{m}$, and ${F}_{b}$ versus the radius $a$ on high-index $({n}_{p}=1.592)$ particles. Other parameters are $\lambda =1.064 \,\upmu{\mathrm{m}}$, ${w}_{0}= 5 \,\mathrm{mm}$*,* $f=5 \,\mathrm{mm}$, $P=4 \,\mathrm{w}$*,* the distance *δz* = 0 from the focus point, and the constant $g=1$.

Two forces are toward light transmission: the scattering force proportional to the intensity of the incident light in the light propagation directions and the gradient force proportional to the intensity gradient along the light gradient direction. Therefore, the second condition is the stability criterion $R$, stating that the backward longitudinal gradient force should be greater than the forward scattering force. According to Einstein’s fluctuation–dissipation theorem, the magnitude of Brownian force can be expressed by

11 $$ F_{b} = \sqrt {12\eta \pi ak_{B} T} . $$

where $\eta =7.977 \times 10-4$ Pa s is the viscosity of water at $T=300 \,\mathrm{K}$^19^*.* Figure 7 demonstrates that the magnitudes of all forces include the maximum axial gradient force ${F}_{Grad,z}^{m}$ at $x,y=0.265 \,\upmu{\mathrm{m}}$, the maximum transverse gradient force ${F}_{Grad,x}^{m}$, the maximum scattering force ${F}_{Scat,x}^{m}$, and the Brownian force ${F}_{b}$ versus the radius $a$ on the high-index microspheres, respectively. The $x$ and $z$ subscripts in each term represent the force component in the coordinate axes. For the high-index particles, when *a* > 4.431 nm, the longitudinal gradient force is greater than the Brownian force in Fig. 7. Therefore, the Brownian force does not affect the particles, and we can stably capture multiple microspheres in the focal plane.

## Conclusion

In this study, we raise and confirm that SMGBs can improve the efficiency of trapping Rayleigh dielectric nanospheres and that SMGBs can simultaneously trap up to four nanospheres on the focal plane due to the unique property of having four individual centers of light intensity distribution. First, we theoretically calculated the Collins integration and a paraxial ABCD optical system. The propagation properties of the electrical field profile and the gradient and scattering forces of focused SMGBs are graphically studied. The study further analyzes the stability criterion and the Boltzmann factor of SMGBs. Based on a case study of trapping particles in water, which undergoes Brownian motion because of thermal fluctuation, the axial gradient and Brownian forces of the SMGBs are compared. The results show that SMGBs can successfully trap Rayleigh dielectric particles with a radius as small as 4.431 nm. The proposed technique of using controllable SMGBs to trap Rayleigh dielectric spheres has a potential for optical micromanipulation. However, applying SMGBs under different conditions must be researched, which will require much work in the future.
